# Fe–Al–Si-Type Iron Aluminides: On the Strengthening by Refractory Metals Borides

**DOI:** 10.3390/ma15207189

**Published:** 2022-10-15

**Authors:** Věra Vodičková, Martin Švec, Pavel Hanus, Šárka Bukovská, Petra Pazourková Prokopčáková

**Affiliations:** 1 Department of Material Science, Faculty of Mechanical Engineering, Technical University of Liberec, 46117 Liberec, Czech Republic; 2 Department of Technology, Faculty of Mechanical Engineering, Technical University of Liberec, 46117 Liberec, Czech Republic

**Keywords:** Fe_3_Al-based cast iron aluminide, titanium, molybdenum and boron addition, grain size, heat treatment, high-temperature yield stress

## Abstract

The effect of boron addition into Fe–28Al–5Si–X (X = -, 2Mo, or 2Ti) on the structure and high-temperature yield stress was investigated. Generally, the alloying of binary Fe_3_Al-type iron aluminides by silicon significantly improves high-temperature mechanical properties by solid-solution strengthening. On the other hand, the workability and ductile properties at room or slightly elevated temperatures get worse with the increasing silicon content. Boron alloying together with titanium or molybdenum alloying is one of the ways to improve the workability of this type of alloy and, at the same time, ensure the formation of a sufficient amount of secondary phase particles required for effective strengthening. In this paper, the influence of 1 at. % of boron on high-temperature yield stress is evaluated in response to structural changes and compared with results obtained previously on the same type of alloy (Fe–28Al–5Si–2X, X= -, Mo, or Ti) but without boron alloying. It can be concluded that the network structure of borides of refractory metals formed due to boron alloying works more effectively for alloy hardening at higher temperatures than a mixture of silicides and carbides present in the boron-free alloy of the same composition.

## 1. Introduction

Iron aluminides belong to the group of intermetallics for structural applications, but their potential has not been fully developed yet. Due to their excellent corrosion resistance, they are considered a material capable of replacing high-alloy steels for high-temperature applications (f.e. brake discs and furnace grids). Unfortunately, too low ductility at room temperature-causing problematic workability-hinders their wider use. The ordered iron aluminides possess low room temperature ductility even though their crystallographic characteristic (for example, more than five independent slip systems) should be the assumption for good. Both Fe_3_Al and FeAl are reasonably ductile at compression, while their tensile ductility is limited at temperatures below brittle-to-ductile transition [1]. The ductility of B2 type Fe40Al alloy can increase with decreasing grain size (<10 μm) [2].

In general, the brittleness of iron aluminides can result from grain boundary properties, and concurrently both Fe_3_Al and FeAl are prone to moisture or hydrogen embrittlement. The reaction with moisture, forming Al_2_O_3_ and atomic hydrogen, and causing brittle cleavage crack propagation, is why the room-temperature ductility is up to 4% maximum in the presence of moisture [3,4,5]. The binary Fe_3_Al alloy shows a cleavage mode of fracture independent of ductility or environment. With aluminum content increasing to 36.5 at. %, the fracture mode is changing from transgranular to intergranular cleavage [1].

If the low ductility of iron aluminides is caused by the environmental effect, it can be improved using alloying. Among others, the effect of chromium on the ductility of iron aluminides was investigated [5]. It is known that small addition of boron can be used to increase the ductility of B2 FeAl [6,7,8]. It was also found that the segregation of boron atoms to the grain boundaries can improve grain boundary cohesion very effectively [9,10].

The mechanism of boron segregation has been intensively studied in FeAl-type intermetallic alloys. There was confirmed that boron segregation has two different origins-non-equilibrium segregation (resulting from an attractive interaction between boron atoms and excess vacancies) working during the quench from high temperature and equilibrium segregation, which is explained by strong repulsive interactions between segregated boron atoms in low-temperature annealed materials. It was also found that bulk properties of FeAl-type iron aluminides are strongly modified by added boron due to the nano-heterogenous structure formation [8]. So, the mechanism of grain boundary strengthening in FeAl-type intermetallic alloys had to be thoroughly investigated on the atomic scale [11,12,13]. A very detailed investigation using a three-dimensional atom probe showed that boron plays a significant role in the formation and stabilization of stacking faults present in the structure of intermetallics. The existence of B-enriched/Al-depleted stacking faults has been demonstrated. This phenomenon of boron enrichment was also observed in Cottrell atmospheres or antiphase boundaries [13]. 

When the amounts of added boron are higher, there was found that this beneficial effect of boron disappears when borides precipitate along grain boundaries [14]. Nevertheless, for Fe_3_Al-based alloys doped with Mo/W–Ti–B was noticed that precipitation of borides at grain boundaries maintained ductility at comparably low temperatures [15,16].

In the case of Fe_3_Al-based iron aluminides, boron addition can also have a similar effect on other mechanical properties (namely ultimate strength). In the past, carbon alloying or boron microalloying together with alloying of the elements having low solubilities in Fe_3_Al-based aluminides had been applied as the most feasible way to achieve precipitation strengthening due to carbides or borides formation [1,17,18,19,20,21].

Currently, the concept of strengthening by incoherent precipitates—borides—has also been successfully developed and applied to improve the high-temperature mechanical properties of Fe_3_Al-based iron aluminides [22,23]. In contrast with carbides, which tend to coarsen rapidly at high temperatures and, due to the shape of particles (plates, needles), can act as sources for crack nucleation, borides can prevent the coarsening of the Fe–Al matrix by pinning grain boundaries [15,24]. Compared to binary D0_3_-ordered iron aluminides, Ti and B-doped aluminides show significantly higher flow stresses and creep resistances. Moreover, the order of the matrix can play an important role-it was shown that the presence of the DO_3_ (L2_1_)-order at higher temperatures increases the alloy strength [15]. 

If the strengthening by borides precipitated in the structure acts together with solid-solution strengthening, the effect of strengthening can be more significant, and the high-temperature characteristics such as creep rate or high-temperature yield stress can be improved.

The article is focused on evaluating the structure and comparison of HT mechanical properties of Fe–Al–Si (-X) intermetallic alloys doped with boron (X = refractory metal). The strengthening by Si soluted in the matrix together with the fine incoherent boron-based precipitates could be beneficial for HT properties of alloy intended for HT applications.

## 2. Materials and Methods

Vacuum induction melting and casting were used for the preparation of the alloys. The nominal chemical composition of the investigated alloys is summarized in Table 1.

Scanning electron microscope (SEM) Tescan Mira 3 was used to study the sample’s microstructure. The microstructure of alloys was studied in the state after oxide polishing. The local chemical composition of phases was determined by Energy Dispersive X-ray Spectroscopy (EDX) using Oxford UltimMax 65 detector. The phase compositions of alloys were verified by X-ray diffraction (XRD) using an X’Pert^3^ Powder diffractometer in Bragg-Brentano geometry (Co Kα radiation, λ = 1.78901 Å). The grain size of alloys and the presence of γ iron were determined using Electron Backscatter Diffraction (EBSD) performed by Oxford Instruments Symmetry detector. The process parameters for each image are described in the manuscript. 

The structure of the three investigated samples was studied in an as-cast state, after heat treatment at 800 ± 5 °C for 100 hrs and after a compression test at 600 °C. The temperature and time of this stabilization annealing were chosen concerning the expected maximum work temperature of this type of material. The equilibrium was reached after annealing at a selected temperature and time. Annealing was carried out in the vacuum furnace.

The values of high-temperature compression yield stress σ_0.2_ were measured by the TESTOMETRIC FS100CT device at temperatures of 20, 600, 700, and 800 °C. The accuracy of the temperature chamber of the device was ±1 °C, and the used initial strain rate was 1.5 × 10^−4^ s^−1^. Both states (as-cast and after heat treatment at 800 °C for 100 h) were tested at all temperatures. Samples for high-temperature tests with dimensions of 6 × 6 × 8 mm were prepared by spark machining.

## 3. Results

### 3.1. Structure of Investigated Alloys

**Figure 1:**
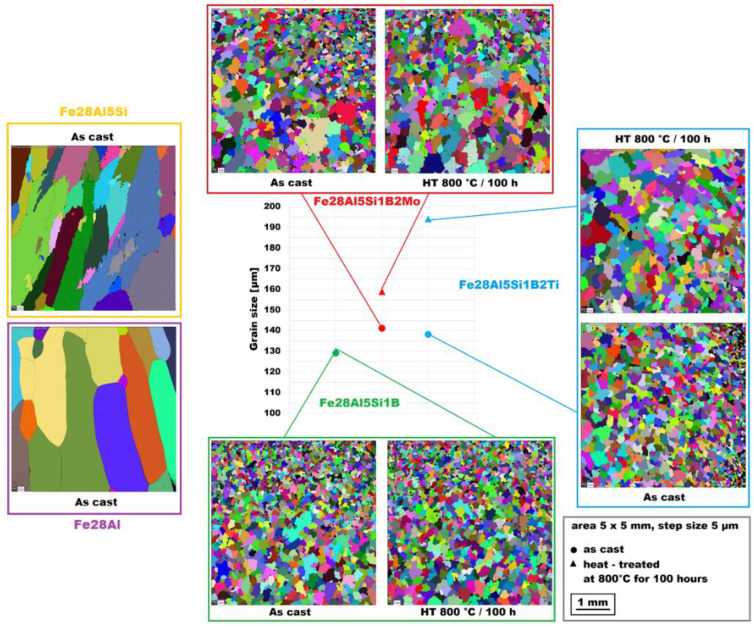
The grain size of investigated alloys in the as-cast state and also after heat treatment at 800 °C for 100 h (the Fe28Al and Fe28Al5Si alloy added for comparison).

All combinations of additives used (boron only, molybdenum–boron, and titanium–boron) caused significant grain refinement (see Figure 1) in comparison to binary Fe–28Al alloy as well as to ternary Fe–28Al5Si alloy, as the grain size of both is usually reported in the order of hundreds of micrometers [25] or of units of millimeters (see Figure 1). The grain size of boron-doped alloys was in the range of 130−140 µm for all investigated alloys in the as-cast state. 

**Figure 2:**
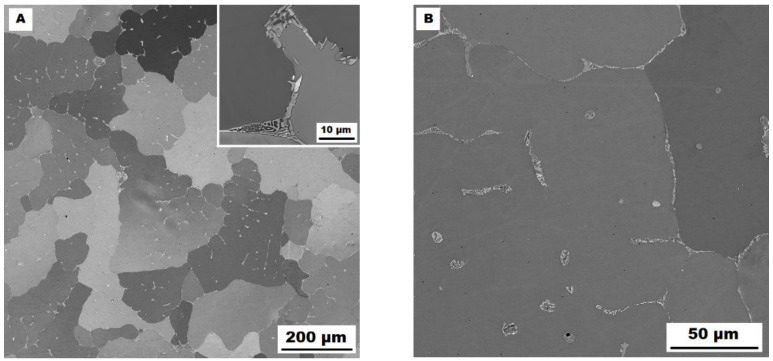
The structure of Fe–28Al–5Si–1B alloy in the as-cast state (SEM, 10 kV, BSE). (**A**) overview of the structure, (**B**) detail of the structure.

**Figure 3:**
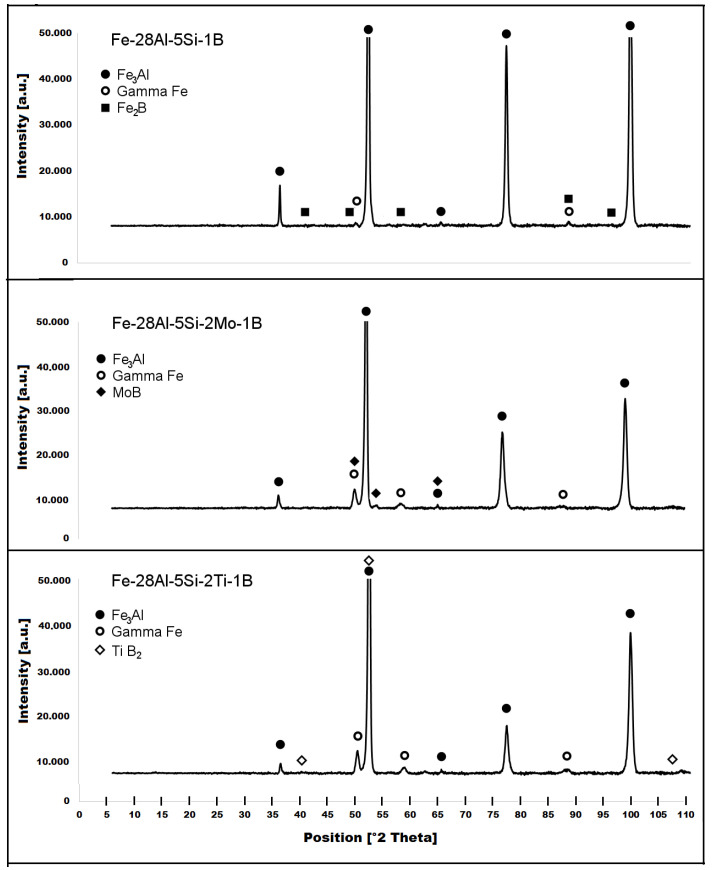
XRD diffractograms of investigated alloys in the as-cast state.

**Figure 4:**
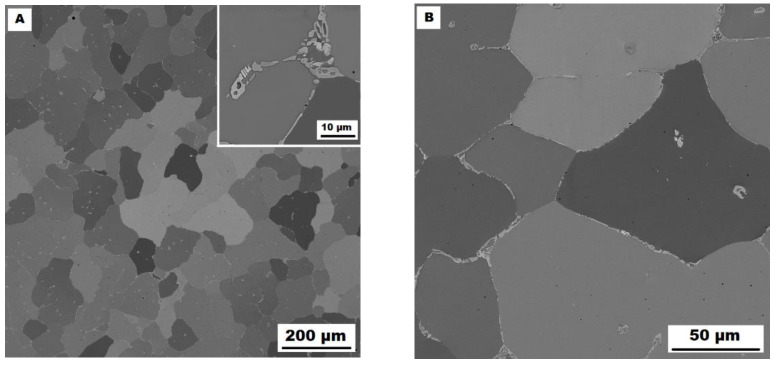
The structure of Fe–28Al–5Si–1B HT 800/100 alloy (SEM, 10 kV, BSE). (**A**) overview of the structure, (**B**) detail of the structure.

The structure of the Fe–28Al–5Si–1B in the as-cast state shows a dendritic character (see Figure 2A). The secondary phase particles are distributed in clusters along dendrite boundaries and also inside dendrites (see Figure 2A,B). This phase was determined by EDX and by XRD as Fe_2_B iron borides (see XRD diffractogram in Figure 3). The stabilization annealing at 800 °C for 100 h (Fe–28Al–5Si–1B HT 800/100 alloy) has no significant effect on the distribution or type of secondary phase (see Figure 4A,B). Additionally, the grain size remains unchanged after annealing (see Figure 1), namely 130 µm for both states.

**Figure 5:**
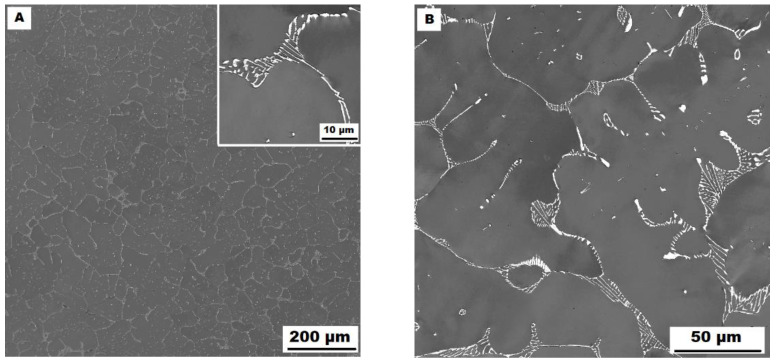
The structure of Fe–28Al–5Si–2Mo–1B alloy in the as-cast state (SEM, 10 kV, BSE). (**A**) overview of the structure, (**B**) detail of the structure.

**Figure 6:**
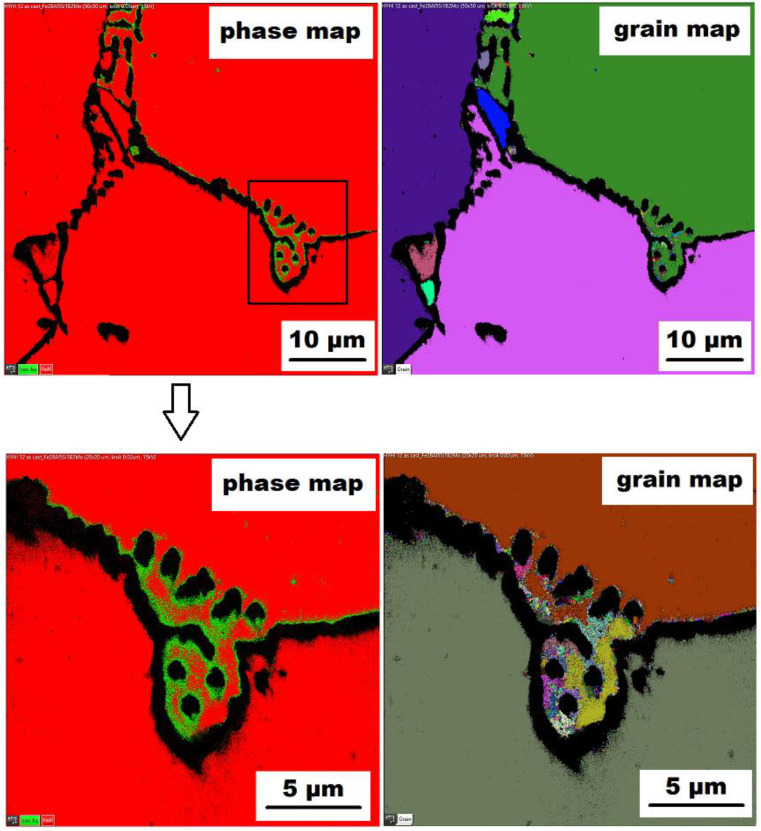
The EBSD phase and grain maps showing the gamma-iron presence in Fe–28Al–5Si–2Mo–1B as-cast alloy (green areas in phase maps), black areas—borides.

**Figure 7:**
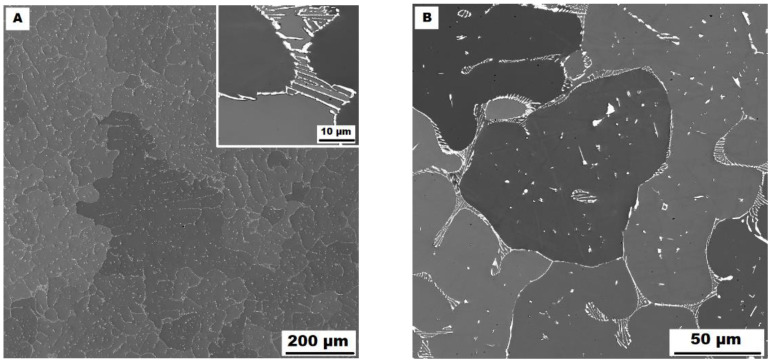
The structure of Fe–28Al–5Si–2Mo–1B HT 800/100 alloy (SEM, 10 kV, BSE). (**A**) overview of the structure, (**B**) detail of the structure.

The character of the structure of Fe–28Al–5Si–2Mo–1B in the as-cast state is also dendritic (see Figure 5A,B). The secondary phase is distributed in the form of the “like-eutectics” (lamellar) areas in the interdendritic spaces and also in the form of the individual fine particles inside the dendrites. Based on EDX and XRD measurements, these particles were identified as MoB molybdenum borides (see Figure 3). It is necessary to notice that the verification of phase by XRD is not quite unambiguous due to the overlapping of phase peaks with Fe_3_Al matrix peaks, but EDX results support this identification. Consistent with XRD analysis, very small areas of gamma-iron were found in the structure using EBSD. This fine-grained phase (grain size in the order of hundreds of nanometers) was distributed very rarely with particles of molybdenum borides, as can be seen in Figure 6. Heat treatment has no significant influence on the stability or distribution of the second phase in the Fe-28Al-5Si-2Mo-1B HT 800/100 alloy (see Figure 7A,B). Only the difference in the grain size was observed-it increases from 141 µm to 159 µm after annealing (see Figure 1).

**Figure 8:**
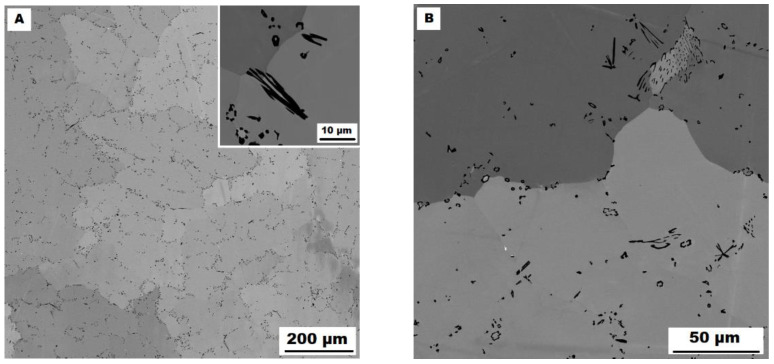
The structure of Fe–28Al–5Si–2Ti–1B alloy in the as-cast state (SEM, 10 kV, BSE). (**A**) overview of the structure, (**B**) detail of the structure.

The dendritic areas are present in the structure of Fe–28Al–5Si–2Ti–1B alloy in an as-cast state (see Figure 8A,B). The secondary phase particles are distributed in the form of needles or oval-shaped “chains” (size range 0.5−20 μm) along dendrite boundaries and also inside dendrites. The precipitates were determined by EDX and XRD as TiB_2_ titanium borides (Figure 3).

**Figure 9:**
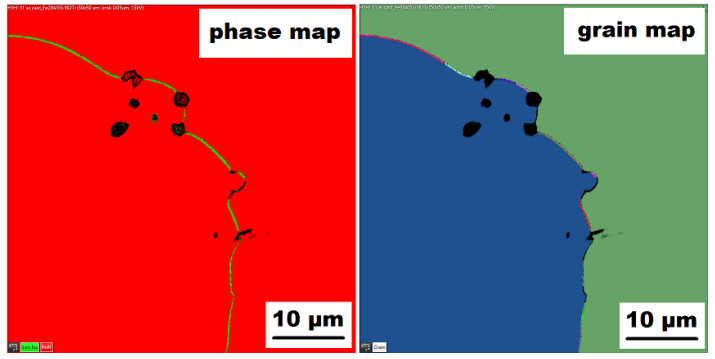
The EBSD phase and grain maps showing the gamma-iron presence in Fe–28Al–5Si–2Ti–1B as-cast alloy (green areas in phase maps).

Similar to the as-cast alloy Fe–28Al–5Si–2Mo–1B, the presence of “nanoscale” areas of gamma-iron (previously identified by X-ray diffraction, Figure 3) was also confirmed by EBSD in the as-cast Fe–28Al–5Si–2Ti–1B alloy, Figure 9.

**Figure 10:**
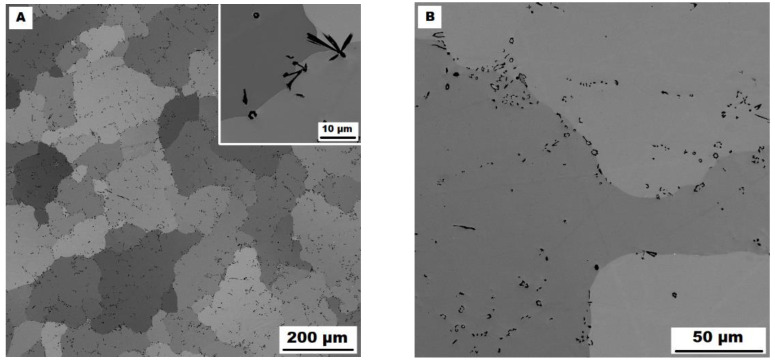
The structure of Fe–28Al–5Si–2Ti–1B HT 800/100 alloy (SEM, 10 kV, BSE). (**A**) overview of the structure, (**B**) detail of the structure.

In contrast to Mo-B doped alloy, these areas were located along the grain boundaries in Ti-B doped alloy, see Figure 9. After heat treatment (alloy **Fe–28Al–5Si–2Ti–1B HT 800/100**), the distribution and type of secondary phase remain without changes (see Figure 10A,B). A significant change was observed in the grain size, which increased from 138 µm to 194 µm after annealing (see Figure 1).

### 3.2. High-Temperature Yield Stress

**Figure 11:**
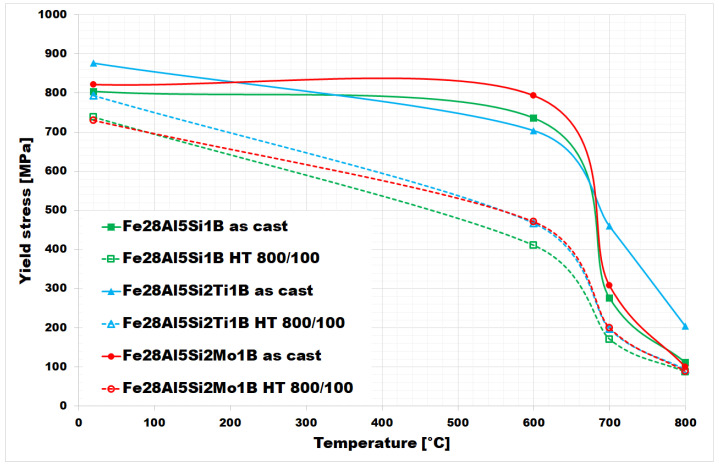
The values of the high–temperature yield stress of investigated alloys (as-cast states and also annealed states).

The values of yield stress in compression at room and elevated temperatures are summarized in Figure 11. They were measured for both the as-cast state and heat-treated state (HT 800/100) of all investigated alloys. In the as-cast state, all three alloys maintain high values of yield stress up to 600 °C (comparable to room temperature values). At 700 °C, the alloy doped with titanium and boron in an as-cast state (full blue line in Figure 11) shows a relatively high value of yield stress in compression (460 MPa). 

It is evident from the chart in Figure 11 that the yield stress values decreased after annealing (HT 800/100) at all tested temperatures for all three investigated alloys.

The difference between yield stress values for as-cast and heat-treated states appears to be most pronounced at 600 °C for Fe–28Al–5Si–1B and Fe–28Al–5Si–2Mo–1B alloys-in the case of Fe–28Al–5Si–2Ti–1B alloy, the maximum decreases in yield stress values are comparable at 600 °C and 700 °C.

In comparison to the same type of alloys doped with titanium or molybdenum only so, without boron [25], the values of yield stress up to 600 °C appear to be of a similar level to these. In the higher temperature range, the yield stress values of Fe–28Al–5Si–2Ti–1B alloy are higher (almost doubled at 800 °C) compared to boron-free alloy in [25].

## 4. Discussion

The relevance of phase equilibria for the strengthening of respective alloys was discussed in detail in [17]. For Fe–Al-type alloys, mechanisms based on strengthening mechanisms derived from the phase diagrams themselves play a significant role, i.e., solid-solution hardening, strengthening by incoherent or coherent particles, and ordering. Nevertheless, grain refinement strengthening through alloying-as shown here-is also available for Fe–Al-based alloys.

Solid-solution strengthening (strengthening by additive atoms) occurs when the presence of an impurity atom in the base metal lattice causes strain in the crystal lattice. An example can be the Fe–Al–Cr system, where within a wide range of concentrations, only solid-solution hardening is possible. 

The strengthening of coherent or non-coherent precipitates is a concept frequently used for increasing mechanical properties, not only in the aluminides field. The precipitates affect the strengthening significantly because they work as dislocation motion obstacles. Principally, the dislocation can overcome the coherent precipitate. The non-coherent precipitate can stop the dislocation motion, but when sufficiently large stress is applied, the dislocation can also overcome this obstacle, i.e., by the Orowan or Friedel mechanism. 

The solubility in solid solution for the third element within the Fe–Al phases is limited in many Fe–Al–X systems, and the possibility of strengthening Fe–Al-based alloys by precipitation of coherent (Fe–Al–Ni system) or non-coherent phase (Fe–Al–Nb, Fe–Al–Zr system) can be used [17].

Additionally, immobile dislocations located in the matrix (dislocation structure includes grain boundaries, subgrains boundaries, and arrangement of dislocations within grains) can be considered effective obstacles. Then the contribution of dislocation strengthening depends on the energy necessary to push dislocation through obstacles of dislocation origin.

For strengthening Fe–Al-based alloys, there is an additional possibility of strengthening by the ordering of crystal structure, e.g., by stabilization of the D0_3_ structure to higher temperatures.

In the case of alloys investigated in this article, the overall strengthening should include the contribution of the solid-solution strengthening by silicon atoms due to the low solubility of silicon in the Fe_3_Al matrix and the contribution of non-coherent precipitates-borides of iron, titanium, or molybdenum. The role of boron could be questionable-although it participates in precipitate formation, its segregation on grain boundaries or on defects (as in the case of Fe–Al-type alloys) is not completely excluded. The strengthening by dislocation arrangement or by grain refinement could be co-operating too. 

According to the simplest/linear model, the additive action of mechanisms could be assumed. However, for Fe–Al-based alloys, the contribution of individual mechanisms to the overall strengthening is not yet completely clear, as there are not as much data available as for other materials. The aspect of strengthening Fe–Al-based alloys at high temperatures is still open and an important issue.

It is evident from Figure 11 that materials with borides of refractory metals (blue and red lines) achieve higher values of yield stress in both states in comparison to alloy Fe–28Al–5Si–1B, except for the value at 600 °C for the as-cast alloys (here the yield strength of Fe–28Al–5Si–1B is higher than that of Fe–28Al–5Si-2Ti–1B, but these values appear to be comparable in the range of measurement errors). This fact could be explained by a noticeably higher density of fine precipitates in Ti/Mo doped alloy structures—compare Figure 2A, Figure 5A and Figure 8A or Figure 4A, Figure 7A and Figure 10A (it has not been quantified). In this case, the contribution of incoherent precipitates—borides—is crucial for high-temperature strengthening, while the effect of solid-solution hardening under the influence of Si atoms dissolved in the matrix should be the same for all investigated alloys. The contribution of solid-solution hardening to the overall strengthening would increase with a higher amount of silicon, as had been shown for silicon/molybdenum doped alloy [26]. The increasing of silicon content led to the enhancement of values of high-temperature yield stress in compression, mainly at temperatures up to 600 °C (the increase of about 160 MPa in the as-cast state)-see [26]. Nevertheless, the strengthening by borides proves to be more advantageous in terms of workability, which deteriorates as the silicon content increases.

**Figure 12:**
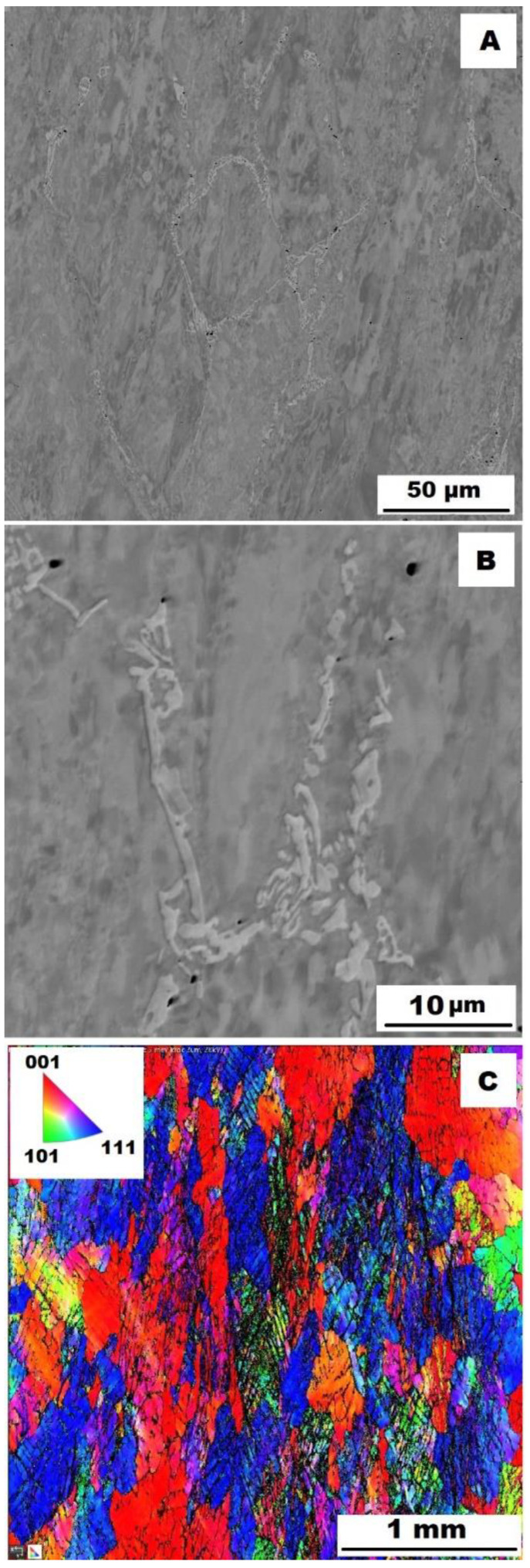
The structure of Fe–28Al–5Si–1B alloy in the as-cast state after compression test at 600 °C. (**A**) Overview of the structure-10 kV, BSE; (**B**) Detail of the structure-10 kV, BSE; (**C**) EBSD map of grains-area 3 × 3 mm, step size 2 µm, 20 kV.

**Figure 13:**
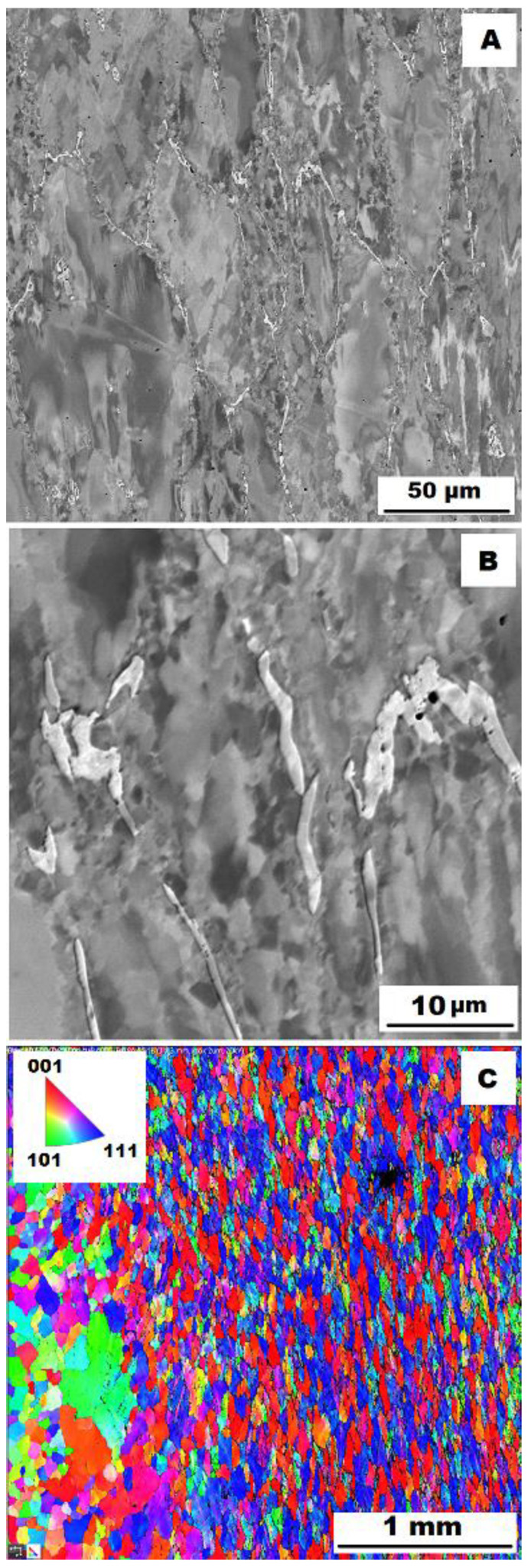
The structure of Fe–28Al–5Si–1B HT 800/100 alloy after compression test at 600°C. (**A**) Overview of the structure-10 kV, BSE. (**B**) Detail of the structure-10 kV, BSE. (**C**) EBSD map of grains-area 3 × 3 mm, step size 2 µm, 20 kV.

**Figure 14:**
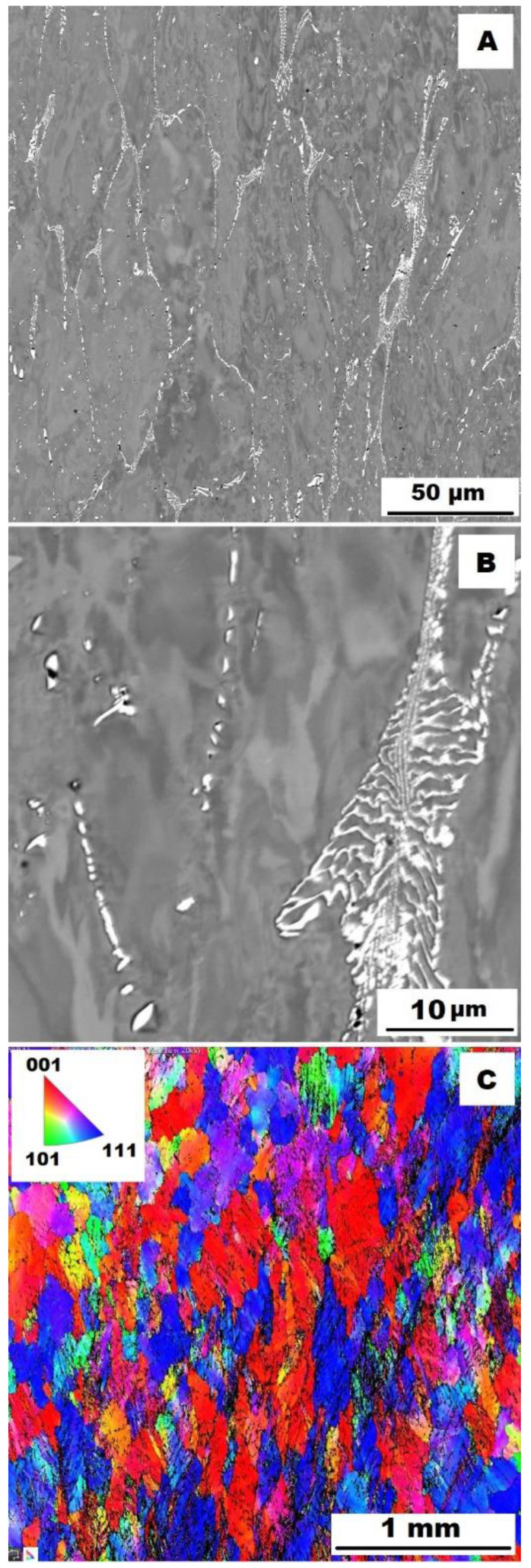
The structure of Fe–28Al–5Si–2Mo–1B alloy in the as-cast state after compression test at 600 °C. (**A**) Overview of the structure-10 kV, BSE. (**B**) Detail of the structure-10 kV, BSE. (**C**) EBSD map of grains-area 3 × 3 mm, step size 2 µm, 20 kV.

**Figure 15:**
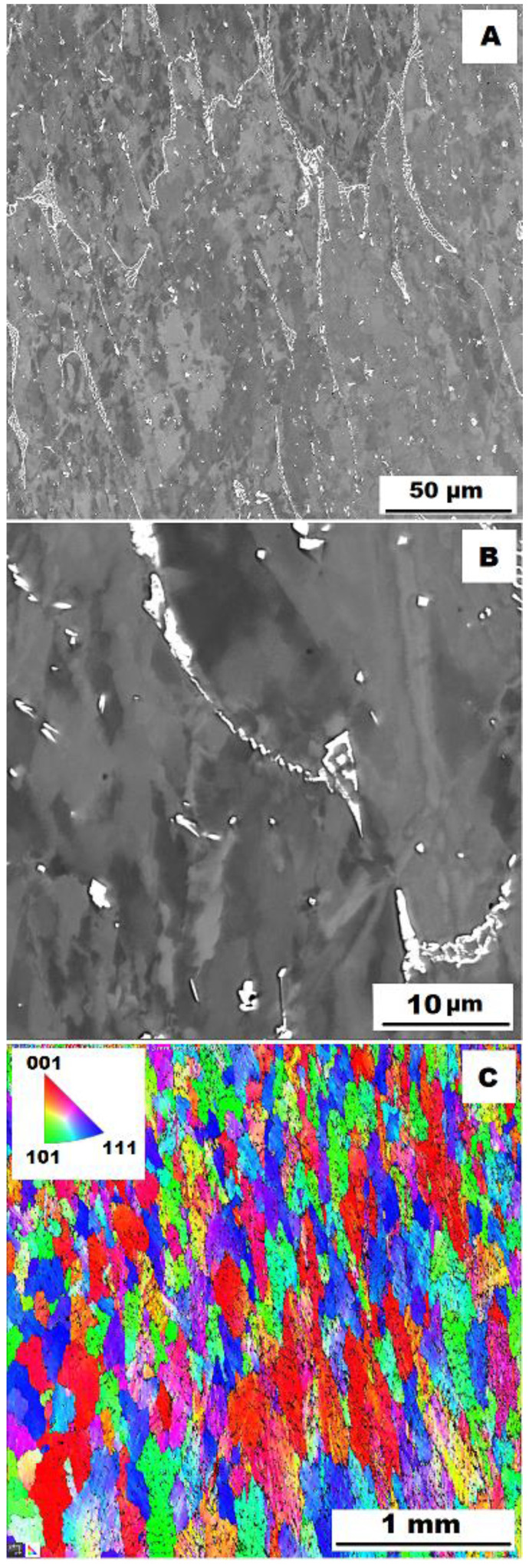
The structure of Fe–28Al–5Si–2Mo–1B HT 800/100 alloy after compression test at 600 °C. (**A**) Overview of the structure-10 kV, BSE. (**B**) Detail of the structure-10 kV, BSE. (**C**) EBSD map of grains-area 3 × 3 mm, step size 2 µm, 20 kV.

**Figure 16:**
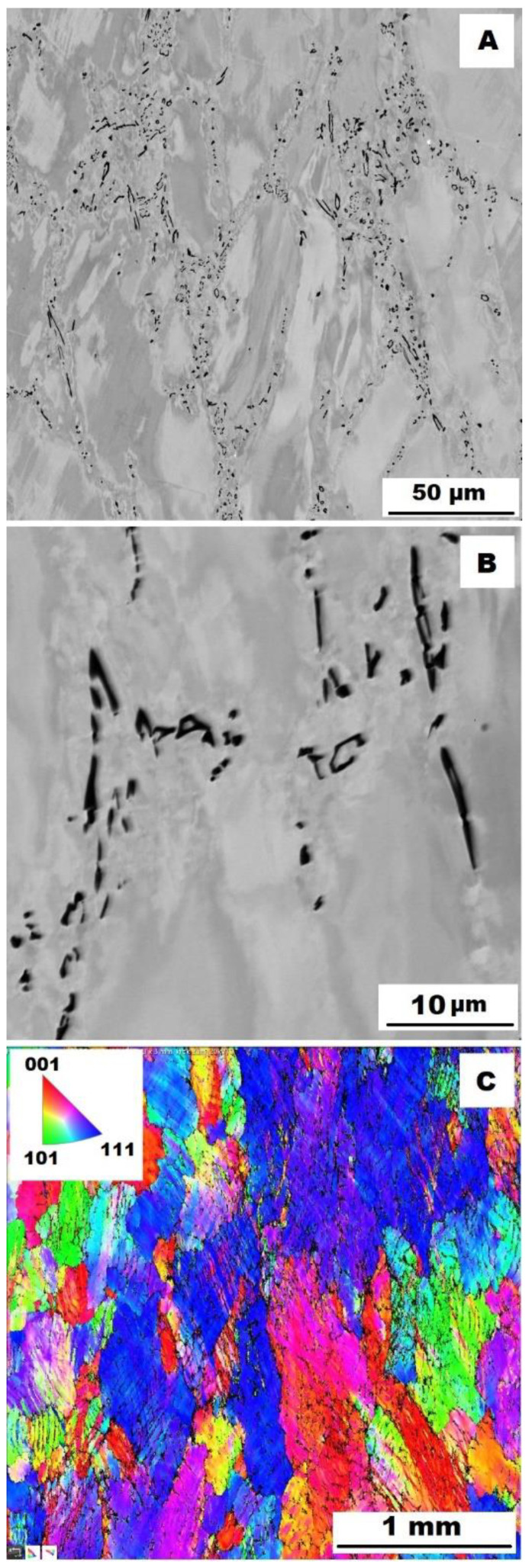
The structure of Fe–28Al–5Si–2Ti–1B alloy in the as-cast state after compression test at 600 °C. (**A**) Overview of the structure-10 kV, BSE. (**B**) Detail of the structure-10 kV, BSE. (**C**) EBSD map of grains-area 3 × 3 mm, step size 2 µm, 20 kV.

**Figure 17:**
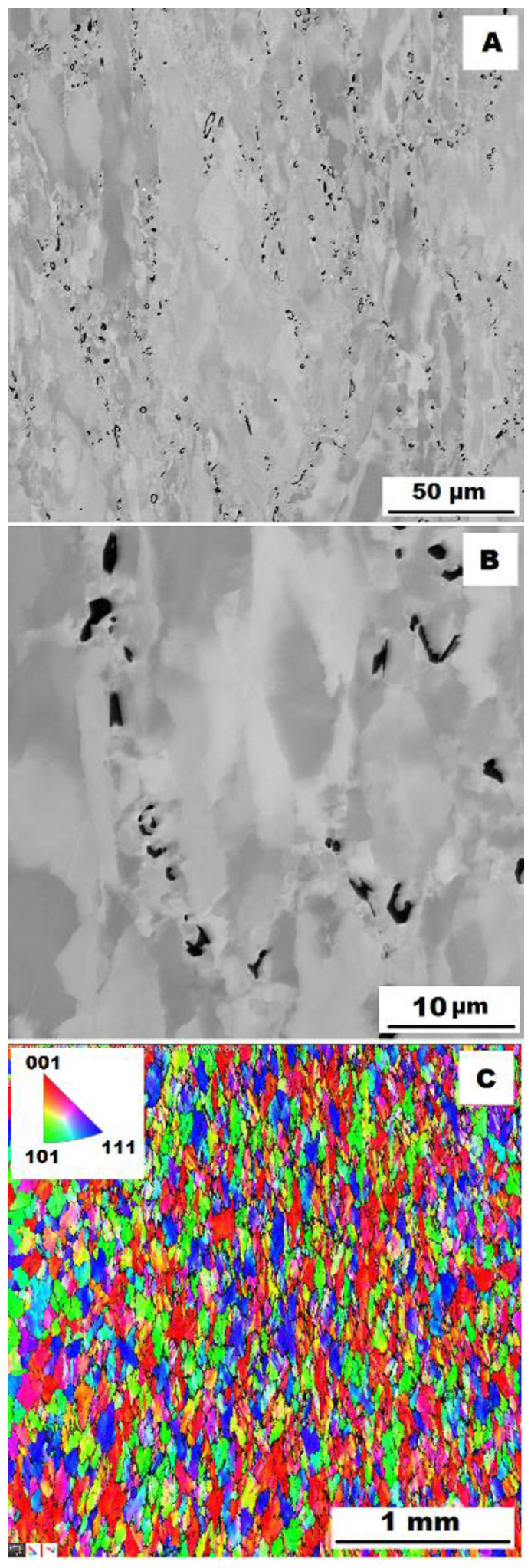
The structure of Fe–28Al–5Si–2Ti–1B HT 800/100 alloy after compression test at 600 °C. (**A**) Overview of the structure-10 kV, BSE. (**B**) Detail of the structure-10 kV, BSE. (**C**) EBSD map of grains-area 3 × 3 mm, step size 2 µm, 20 kV.

To assess the strengthening mechanisms interactions during deformation, the samples deformed at 600 °C were subjected to structure- and EBSD analysis. The structures of samples after compression tests at 600 °C are shown in Figure 12, Figure 13, Figure 14, Figure 15, Figure 16 and Figure 17. There can be stated from the comparison of EBSD Figures for as-cast and annealed states (compare Figure 12C to Figure 13C; Figure 14C to Figure 15C and Figure 16C to Figure 17C) that the annealed samples (800 °C/100 h) have recrystallized during compression tests at 600 °C. On the other hand, the samples in the as-cast state show a deformation texture (see EBSD Figures). In both cases (in as-cast and annealed states), very small grains started to grow in narrow areas near the secondary particles. This phenomenon is more pronounced in as-cast states of samples—compare Figure 12A,B to Figure 13A,B; Figure 14A,B to Figure 15A,B, and Figure 16A,B to Figure 17A,B.

The decrease in yield stress values of alloys after annealing corresponds to Figure 12, Figure 13, Figure 14, Figure 15, Figure 16 and Figure 17 very well. The deformed structure of as-cast samples shows higher values of yield stress due to a higher density of dislocations and, therefore, a higher stress level in the crystal lattice-the deformation strengthening is working. In annealed samples, recrystallization has taken place, so dislocation recovery has caused a loss of strengthening.

In both states, the formation of very small grains along phase boundaries could contribute to strengthening. In the as-cast state, the volume fraction of these areas is higher.

In sum, the structure recrystallized during compression tests, together with a lower amount of very small recrystallized grains near the secondary particles, can cause lower values of yield stress in the case of samples annealed at 800 °C for 100 h. 

As far as the presence of gamma iron in the structure of investigated alloys, a similar phenomenon has only been described exceptionally for Fe–Al-type intermetallic alloys. In Fe–Al-type intermetallics manufactured by the SHS process, only the temporary presence of gamma iron during reactive sintering was confirmed using the in-situ XRD study of the formation of intermetallic phases [27]. 

The above-mentioned presence of gamma iron should be due to the limited diffusion of aluminum. There can be several reasons for this phenomenon. In Figure 6 (grain map-detail), the formation of superfine grains can be observed in the areas surrounding the “chain” of borides (or between them). The higher density of grain boundaries (and therefore the higher density of crystal lattice defects) in the areas close to boride chains should help the diffusion of Al atoms. Nevertheless, the limitations of aluminum atoms diffusion could be connected with boron alloying-as stated above (in the Introduction), it was confirmed in Fe–Al-type iron aluminides that boron atoms can segregate on grain boundaries, phase boundaries, or stacking faults. It is not excluded that a similar phenomenon may also occur in Fe_3_Al-type iron aluminides. Provided that the major part of added boron is drained for borides formation, and the remaining small amount of boron tends to nanosegregation along with the interface, then boron atoms maybe preferentially occupy these grain boundaries (or defects) and deteriorate their “permeability” for diffusing atoms, thereby supporting the formation of aluminum-depleted submicron regions.

## 5. Conclusions

For Fe–Al–Si–X alloys, it has been verified that the addition of boron effectively prevents grain coarsening that occurs as a result of annealing. 

The Ti-doped as-cast alloy reached significantly higher yield stress values at 700 and 800 °C compared to the Fe-28Al-5Si-1B alloy due to the formation of an almost continuous network structure of titanium borides. After annealing (HT 800/100), slightly higher yield stress values are achieved for both alloys doped with Ti or Mo at all tested temperatures. 

Compared to the same alloys without boron [25] can be stated that the presence of borides in the structure of Ti and B doped alloy enhances high-temperature yield stress values in compression in higher temperatures, especially in the temperature range of 700°−800°C. So, in the case of Fe–28Al–5Si–2Ti–1B alloy, a continual network structure of titanium borides is more effective for alloy strengthening than a mixture of silicides and carbides in the form of residual eutectics-like areas for boron-free alloy [25]. 

During compression tests, the recrystallization occurs for all tested alloys annealed at 800°C for 100 h. Lower yield stress values in the case of annealed alloys are related to the recovery of dislocations due to the recrystallization during deformation. In both states—as-cast and annealed—the formation of regions of very small grains along the phase interfaces was noted during deformation, which may also contribute to strengthening.

## Figures and Tables

**Table 1:** The nominal chemical composition of investigated alloys [at. %].

Alloy	Fe	Al	Si	Mo	Ti	B
Fe–28Al–5Si–1B	Bal.	28	5	-	-	1
Fe–28Al–5Si–2Mo–1B	Bal.	28	5	2	-	1
Fe–28Al–5Si–2Ti–1B	Bal.	28	5	-	2	1

## Data Availability

Not applicable.
